# Accelerated intermittent theta burst stimulation in major depression induces decreases in modularity: A connectome analysis

**DOI:** 10.1162/netn_a_00060

**Published:** 2018-11-01

**Authors:** Karen Caeyenberghs, Romain Duprat, Alexander Leemans, Hadi Hosseini, Peter H. Wilson, Debby Klooster, Chris Baeken

**Affiliations:** School of Psychology, Faculty of Health Sciences, Australian Catholic University, Sydney, Australia; Department of Psychiatry and Medical Psychology, Ghent University, Ghent, Belgium; Image Sciences Institute, University Medical Center Utrecht, Utrecht, The Netherlands; Department of Psychiatry and Behavioral Sciences, School of Medicine, Stanford University, Stanford, CA, USA; School of Psychology, Faculty of Health Sciences, Australian Catholic University, Sydney, Australia; Eindhoven University of Technology, Department of Electrical Engineering, Eindhoven, The Netherlands; Academic Center for Epileptology Kempenhaeghe, Heeze, The Netherlands; Department of Psychiatry and Medical Psychology, Ghent University, Ghent, Belgium; Department of Psychiatry, University Hospital UZBrussel, Brussels, Belgium; and Ghent Experimental Psychiatry (GHEP) Lab, Ghent, Belgium

**Keywords:** Structural connectivity, Brain stimulation, Depression, Diffusion MRI, graph theory

## Abstract

Accelerated intermittent theta burst stimulation (aiTBS) is a noninvasive neurostimulation technique that shows promise for improving clinical outcome in patients suffering from treatment-resistant depression (TRD). Although it has been suggested that aiTBS may evoke beneficial neuroplasticity effects in neuronal circuits, the effects of aiTBS on brain networks have not been investigated until now. Fifty TRD patients were enrolled in a randomized double-blind sham-controlled crossover trial involving aiTBS, applied to the left dorsolateral prefrontal cortex. Diffusion-weighted MRI data were acquired at each of three time points (T_1_ at baseline; T_2_ after the first week of real/sham aiTBS stimulation; and T_3_ after the second week of treatment). Graph analysis was performed on the structural connectivity to examine treatment-related changes in the organization of brain networks. Changes in depression severity were assessed using the Hamilton Depression Rating Scale (HDRS). Baseline data were compared with 60 healthy controls. We observed a significant reduction in depression symptoms over time (*p* < 0.001). At T_1_, both TRD patients and controls exhibited a small-world topology in their white matter networks. More importantly, the TRD patients demonstrated a significantly shorter normalized path length (*p*_*AUC*_ = 0.01), and decreased assortativity (*p*_*AUC*_ = 0.035) of the structural networks, compared with the healthy control group. Within the TRD group, graph analysis revealed a less modular network configuration between T_1_ and T_2_ in the TRD group who received real aiTBS stimulation in the first week (*p* < 0.013). Finally, there were no significant correlations between changes on HDRS scores and reduced modularity. Application of aiTBS in TRD is characterized by reduced modularity, already evident 4 days after treatment. These findings support the potential clinical application of such noninvasive brain stimulation in TRD.

## Author Summary

Accelerated noninvasive neurostimulation has shown promise to rapidly improve clinical symptoms in patients suffering from treatment-resistant depression. However, the stimulation effects on brain networks have not been well investigated but may be necessary to improve clinical outcome. To examine treatment-related changes in the organization of brain networks, graph analysis was performed on structural connectivity in 50 treatment-resistant depressed patients which underwent such a stimulation protocol. Compared to nondepressed individuals, depressed patients displayed less structural integration, especially in more distal networks of the brain. More densely interconnected regions, especially when actively stimulated, may be of essence to explain the clinical improvement, already present after 4 days of accelerated neurostimulation.

## INTRODUCTION

Major depressive disorder (MDD) is a worldwide mental health problem (WHO) and is characterized by affective, cognitive, and somatic symptoms impeding the daily life and activities of the patient. MDD typically manifests as a chronic condition, characterized by a relapsing/remitting course and by severe impairment that persists even during periods of remission (Conradi et al., 2011). Moreover, 20–30% of patients with MDD fail to respond to antidepressant medication and/or psychotherapy, a condition referred to as treatment-resistant depression (TRD) (Vieta & Colom, 2011; van Randenborgh et al., 2012; Trevino et al., 2014). As such, TRD is associated with significantly greater medical costs and productivity loss than treatment-responsive forms, highlighting the need for more effective strategies.

Noninvasive brain stimulation methods have shown promise for improving clinical outcomes in patients suffering from TRD (for reviews, see Lefaucheur et al., 2014; Brunoni et al., 2017). The majority of studies using repetitive transcranial magnetic techniques (rTMS) for clinically depressed patients have already shown that a series of daily sessions of high-frequency (HF)-rTMS delivered on the left dorsolateral prefrontal cortex (DLPFC) or low-frequency (LF)-rTMS applied to the right DLPFC are effective in reducing depressive symptoms (Lefaucheur et al., 2014). Furthermore, it has been stated that theta burst stimulation (TBS)—a specific rTMS protocol that uses bursts of high-frequency stimulation at repeated intervals—may result in superior clinical outcomes (Huang et al., 2005; Plewnia et al., 2014; Li et al., 2014). More recent studies have examined whether accelerated stimulation paradigms can not only yield higher response rates but also reduce the total time of treatment, with promising results (Holtzheimer et al., 2010; Baeken et al., 2013) For example, in our recent randomized, sham-controlled crossover accelerated intermittent theta burst (aiTBS) study in TRD patients (Desmyter et al., 2016; Duprat et al., 2016), we showed significant (acute) reductions in depression severity symptoms and suicide ideation. Despite these promising results, the underlying neurobiological mechanisms supporting these treatment-related changes remain unclear. Insight into these mechanisms may inform our understanding of the neurobiological bases of depression and our ability to target those brain systems to optimize treatment. Indeed, brain stimulation methods hold promise in selectively modulating the activity of neuronal networks that may be implicated in depression, improving clinical outcomes (Huang et al., 2005; Sale et al., 2015). In an influential resting-state functional connectivity paper, Fox et al. (2012) showed specific connectivity patterns in relation to clinical rTMS treatment outcome between the (subgenual) anterior cingulate and prefrontal cortices. Globally, these observations have been replicated by others, stimulating the left DLPFC (Baeken et al., 2014, 2017; Liston et al., 2014; Philip et al., 2018) as well as targeting the dorsomedial prefrontal cortex (DMPFC) (Salomons et al., 2014). Furthermore, stimulating the DLPFC causes network-specific increases in functional connectivity in similar regions also in healthy individuals (Tik et al., 2017). In an effort to personalize rTMS treatment, Drysdale et al. (2017) defined four neurophysiological depression subtypes (“biotypes”) characterized by distinct patterns of dysfunctional connectivity in limbic and frontostriatal networks, responsive or not to DMPFC rTMS treatment. Although functional connectivity alterations are associated with the pathophysiology of MDD, future research is needed to investigate how changes in such abnormal patterns of fluctuating communication may contribute to successful treatment of this severe psychiatric illness (Brakowski et al., 2017; Kaiser et al., 2015).

A connectome framework may further improve our understanding of the biological mechanisms of therapeutic effects. Recently, graph theoretical analyses of structural and functional brain connectivity in humans have contributed to new conceptualizations of the pathogenesis of MDD (for a review, see Gong & He, 2015). For example, using resting-state fMRI, Zhang et al. (2011) found that the drug-naive, first-episode MDD patients (*N* = 30) showed lower path length, higher global efficiency, and increased nodal centralities. In another functional connectome study, Chen et al. (2017) found decreased clustering coefficient, local efficiency, and transitivity in MDD patients (*N* = 16). The results of Guo et al. (2012) revealed abnormal nodal centralities in resting-state functional brain networks of 38 MDD patients compared with healthy controls. Using gray matter covariance networks, Singh et al. (2013) demonstrated significantly decreased clustering coefficient and nodal alterations in patients with MDD (*N* = 93) compared with healthy controls. Twenty-nine MDD participants showed changes (mainly in cognitive-emotional circuitry and fronto-parietal circuitry) in eigenvector centrality, local clustering coefficient, and nodal efficiency in the study by Qin et al. (2014) using white matter networks. In early stage MDD patients, a significant decrease in small-worldness and a significantly decreased strength in the frontal-subcortical and limbic regions was found by Lu et al. (2017). Korgaonkar et al. (2014) found no significant group differences for the graph theory measures, despite the fact that their network-based statistics revealed lowered structural connectivity in two subnetworks in a cohort of 95 MDD outpatients. In sum, these connectome studies have revealed that patients with MDD are associated with anomalies in the topological organization of large-scale functional and structural brain networks (involving global integration, local segregation, modular structure, and network hubs).

To date, only a few studies have examined the effects of stimulation on brain networks in humans, but these studies mainly used transcranial direct-current stimulation or deep brain stimulation treatments. Specifically, two studies have used a connectome approach to examine the effects of brain stimulation by using transcranial direct-current stimulation in healthy volunteers (Polanía et al., 2011; Peña-Gómez et al., 2012), albeit studies of functional connectivity. In addition, a recent structural connectivity study in 11 TRD patients (Riva-Posse et al., 2017) examined the effects of subcallosal cingulate deep brain stimulation. Their results supported the advantage of using an individualized tractography map that is based on a group “connectome blueprint” of past responders to prospectively identify the implantation target, surpassing traditional approaches that rely on anatomical landmarks or stereotactic coordinates. However, their connectome analysis included only four white matter bundles (i.e., forceps minor, uncinate fasciculus, cingulum, and fronto-striatal fibers). Furthermore, whether and how aiTBS might produce measurable and durable changes in structural brain integration and segregation in TRD is unclear. Building on our previous clinical studies of aiTBS (Desmyter et al., 2016; Duprat et al., 2016), our current exploratory study examined whether an aiTBS protocol could induce changes in the organizational properties of brain networks and whether such changes are associated with amelioration of depressive symptoms.

## METHODS

### Participants

This study was part of a larger project investigating the effects of aiTBS on depressive symptoms and suicide risk (http://clinicaltrials.gov/show/NCT01832805). The study was carried out in accordance with the principles of the Declaration of Helsinki and approved by the local ethics committee of the University Hospital Ghent. Written consent was obtained from all subjects.

In the present study, a total of 106 adults aged 18 to 65 years (mean age = 39.9 years, *SD* = 12.2 years, 40 men and 66 women) were included. Sixty healthy controls (mean age = 38.6 years, *SD* = 12.5 years, 26 men) were recruited from the general population with flyers. Volunteers received payment for their participation. By using the structured Mini-International Neuropsychiatric Interview (MINI), 46 patients were diagnosed with major depression (mean age = 41.6 years, *SD* = 11.7 years, 14 men and 32 women); the MINI is a short, accurate structured interview for DSM-IV and ICD-10 psychiatric disorders for clinical trials. Patients were at least stage I therapy-resistant depressed according to the Thase and Rush staging model (Rush, Thase, & Dubé, 2003), that is, patients had not responded to at least one antidepressant pharmacotherapy trial. Participants with contraindications to MRI scanning (e.g., ferrous implant, claustrophobia, and pacemaker), bipolar or psychotic symptoms, history of epileptic insult, cerebral surgery, alcohol dependence, or a suicidal attempt within 6 months were excluded. Antidepressant and antipsychotic medication and mood stabilizers were gradually tapered off and fully stopped 2 weeks before the start and during the whole period of the aiTBS treatment. Healthy volunteers were free of mental diagnosis (also assessed with the MINI) and any psychotropic agent.

### Brain Stimulation Protocol

All patients were enrolled in a randomized, double-blind, sham-controlled crossover study (for an overview of the design, see Supporting Information Figure S1, Caeyenberghs, Duprat, Leemans, Hosseini, Wilson, Klooster, & Baeken, 2018). Patients were randomly allocated to two groups: during the first week, one group received the active (verum) stimulation and the other group started with the sham condition. The treatment conditions (sham, real) were reversed during the second week. The healthy control group only underwent baseline measurements and did not receive any stimulation. The interested reader is also referred to our previous studies (Desmyter et al., 2016; Duprat et al., 2016).

Intermittent TBS stimulation was applied using a Magstim Rapid2 Plus1 magnetic stimulator (Magstim Company Limited, Wales, UK) with a figure-of-eight-shaped coil. A stimulation intensity of 110% of the patient’s resting motor threshold was administered during treatment. We used the Brainsight neuronavigation system (BrainsightTM, Rogue Research) to identify the site of stimulation (i.e., the center part of the midprefrontal gyrus [Brodmann 9/46]) based on the anatomical MRI scan of each individual in order to accurately target the left dorsolateral prefrontal cortex (DLPFC).

aiTBS was delivered at five sessions per day during 4 days. Between the daily sessions there was a pause of approximately 15 min. Each aiTBS session consisted of 54 trains of 10 bursts of three stimuli. These stimuli were applied in a 50 Hz frequency: the bursts were repeated every 200 ms. This resulted in 2 s of stimulation with a cycling period of 8 s, yielding 1,620 stimuli per session. With a total of 20 sessions, this yielded a sum of 32,400 stimuli per complete treatment. For the sham condition, a specially designed sham coil, identical in form and sound to the active coil but without delivering any active stimulation, was placed on the same target site. The aiTBS administrators could not be blinded to assignment as the physical coils needed to be changed. Throughout the whole treatment (aiTBS and sham), patients were blindfolded, wore earplugs, and were kept unaware of the type of stimulation.

To examine depression severity changes, the Hamilton Depression Rating Scale (HDRS; Hamilton, 1967) was administered at three time points (i.e., at baseline [T_1_], after the first week of stimulation [T_2_], and after the second week of stimulation [T_3_]) by an independent rater, blind to the treatment condition.

### MRI Data Acquisition

MR examination was performed on a Siemens 3T TrioTim MRI scanner (Siemens, Erlangen, Germany) by using a 32-channel head coil at the Ghent Institute of Functional Imaging (University of Ghent). Diffusion images were acquired using a single-shot echo planar imaging (EPI) sequence. The major acquisition parameters included the following: repetition time (TR) = 8,500 ms, echo time (TE) = 85 ms, voxel size = 2.0 × 2.0 × 2.0 mm^3^, slice thickness = 2 mm, field of view (FOV) = 244 × 244 mm^2^, matrix size = 122 × 122, 68 contiguous sagittal slices, no gap, scan time = 9:14 min). For each participant, a total of 62 diffusion-weighted images (DWI) were acquired, including two non-diffusion-weighted images (b = 0 s/mm^2^) and 60 diffusion-weighted images (b = 800 s/mm^2^) with 62 noncollinear gradient directions. In addition, we acquired anatomical scans using a 3D-TFE sequence (TR/TE = 2,530 ms/2.58 ms; flip angle = 7 deg; FOV = 220 × 220 mm^2^; resolution = 0.9 × 0.9 × 0.9 mm^3^; number of slices = 176; TA = 6 min). Of note, scans of the patients were administered at three time points (T_1_ at baseline, T_2_ after the first week of real/sham aiTBS stimulation, and T_3_ after the second week of treatment), while only baseline scans were collected from the healthy control group.

### MRI Preprocessing

**Figure 1.**
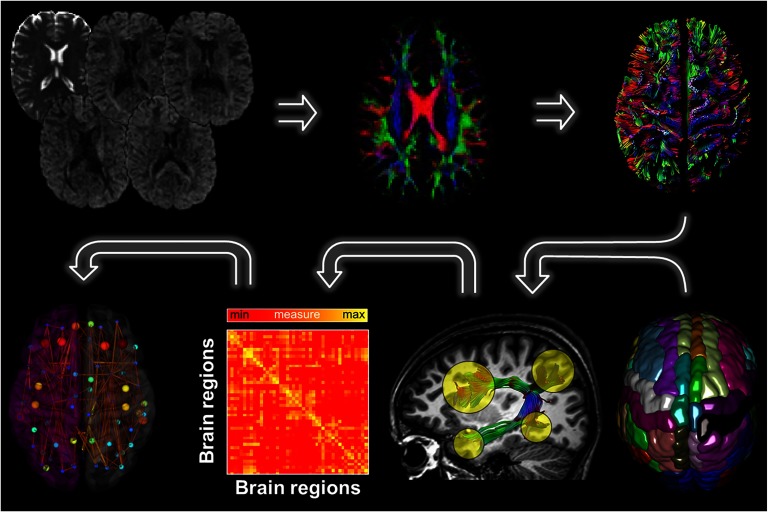
Overview of the MRI data processing pipeline. First, for each DWI dataset a whole brain deterministic tractography was performed using ExploreDTI. The Desikan-Killiany atlas, consisting of 89 brain regions, was then used to segment the fiber bundles between each pair of ROIs. We next determined the density weight between each pair of regions, resulting in 89 × 89 connectivity matrices. Finally, from the resulting brain network graph metrics were computed.

Figure 1 shows the DWI and T1 processing pipeline. FreeSurfer (http://surfer.nmr.mgh.harvard.edu) was used for cortical reconstruction and volumetric segmentation reconstruction of the brain’s surface by using a semiautomated approach described in detail elsewhere (Fischl et al., 2002; Jovicich et al., 2009), with the use of additional computing resources from the Multi-modal Australian ScienceS Imaging and Visualisation Environment (MASSIVE) cluster at Monash University (https://www.massive.org.au/; Goscinski et al., 2014). Images were processed automatically using the FreeSurfer longitudinal stream (Reuter & Fischl, 2011). Default parameters were used for all processing steps. The results for each subject at each time point were carefully inspected to ensure the accuracy of the skull stripping, segmentation, and cortical surface reconstruction. Poor data quality, such as inclusion of dura in the pial surface after skull stripping, and surface deformations, was revealed in two TRD patients. These T1 datasets were excluded from all further analyses. Finally, the T1.mgz (i.e., the FreeSurfer T1 image) and aparc+aseg.mgz (i.e., image containing ROIs constructed by the FreeSurfer pipeline) files were converted to NIfTI format (T1.nii and aparc+aseg.nii) to be used in further diffusion analyses.

ExploreDTI (v4.8.6) (Leemans et al., 2009) was used to process each DWI dataset by using the following multistep-procedure: first, the FreeSurfer T1.nii files were processed using the mask function from ExploreDTI, applying a kernel size of morphological operators of 5 and a threshold of 0.05. Subsequently, diffusion data were corrected for signal drift, subject motion, eddy current-induced distortions, and susceptibility artifacts (Irfanoglu et al., 2012; Leemans & Jones, 2009; Vos et al., 2017), with the masked T1.nii files as undistorted (target) scans. The corrected diffusion results were quality checked in every subject. Poor data quality was observed in one patient because of severe head motion (exceeding the size of 1 voxel), and two patients because of artifacts. These DWI data were excluded from further analyses.

The diffusion tensor was estimated from the corrected images with the robust fitting routine REKINDLE (Veraart et al., 2013; Tax et al., 2015). To correct for EPI distortions, the DWI were nonrigidly aligned (image contrast during registration is the FA) to the subjects’ individual high-resolution T1-weighted image, with the deformation field constrained along the phase encoded A-P axis. Whole-brain tractography was reconstructed in the individual T1 space (Basser et al., 2000) with a uniform seed point resolution of 2 mm^3^, step size of 1 mm, an angle threshold of 30°, and FA threshold of 0.2.

### Connectome Analyses

The Desikan–Killiany atlas was also used to derive the nodes for our graph theoretical analyses, resulting in 89 ROIs in each subject (see Supporting Information Table S1, Caeyenberghs et al., 2018). These comprised all cortical ROIs from the Desikan–Killiany atlas (60 cortical areas), plus cerebellum cortex, thalamus proper, caudate, putamen, pallidum, hippocampus, amygdala, accumbens-area and ventral diencephalon (all of them bilateral), and brainstem. Interregional connectivity was then examined by determining the connection density (number of fiber connections per unit surface and normalized for fiber bundle length) between any two nodes (i.e., any two regions of the Desikan–Killiany template; Hagmann et al., 2008). The resulting density weight was converted to symmetrical connectivity matrices (89 × 89 ROIs) and the main diagonal was set to zeros. These matrices were subsequently used for graph theoretical analysis, as discussed in the next section.

### Graph Theoretical Analyses

Analyses of network properties were performed using the Graph Analytical Toolbox version 1.4.1 (GAT; Hosseini et al., 2012a), which uses routines of the Brain Connectivity Toolbox for network metrics calculation (Rubinov & Sporns, 2010).

### Cross-Sectional Graph Theoretical Analysis

#### Threshold selection.

To allow comparison of global network properties between groups and avoid biases associated with using a single threshold (van Wijk et al., 2010), the matrices were thresholded at a range of network densities (Dmin: Dmax) (Bassett et al., 2008; Bernhardt et al., 2011; He et al., 2008; Hosseini et al., 2012a, 2012b). Where Dmin was defined as the minimum density above which both of the networks were not fragmented (0.10 for this study), and Dmax was set at 0.20 as after this threshold the graphs became increasingly random.

#### Network metrics.

For each threshold, the following global network metrics were extracted: small-worldness, normalized clustering coefficient, normalized shortest path length, global efficiency, clustering coefficient, modularity, and assortativity. Additionally, the following four regional network metrics were calculated for each threshold: degree, local efficiency, node betweenness centrality, and clustering coefficient. An explanation for each global and regional network metric can be found in Supporting Information Table S2 (Caeyenberghs et al., 2018). All network metrics were compared with the corresponding values obtained and averaged from 20 random networks with the same number of nodes, edges, and degree distribution (Hosseini & Kesler, 2013).

#### Group comparisons global network metrics.

Nonparametric permutation testing (5,000 repetitions) was used to determine the statistical significance of between-group differences, controlling for age (He et al., 2008; Hosseini et al., 2012b). In each permutation, the connectivity matrices of each participant were randomly reassigned to one of the two groups (TRD, controls) so that each randomized group had the same number of subjects as the original groups. Then, an association matrix was obtained for each randomized group. These association matrices were then normalized, and network measures were calculated for each network at each density and summarized using area under the curve (AUC) (Hosseini et al., 2012b). This resulted in a null distribution of differences, against which the *p* values of the actual differences in the curve functions obtained by comparing controls and TRD patients were computed. This nonparametric permutation test based on AUC inherently accounts for multiple comparisons across the range of densities (Bassett et al., 2011; Singh et al., 2013).

#### Group comparisons regional network metrics.

The same permutation procedure was used to test the significance of the between-group differences in regional network measures, that is, comparing the AUC of the regional network measures over the specified density range. The *p* values reported for regional differences between groups were false discovery rate (FDR) corrected for multiple comparisons, with a statistical threshold of *p* < .05.

### Hub Analysis

Finally, we also performed a qualitative hub analysis. The nodes with the largest betweenness centrality were considered to be the most important regions in the brain network (hubs). Hubs are essential for coordinating brain functions through their connectivity with various brain regions (Cole et al., 2010) and facilitate efficient communication across the network. In the present study, a node was considered to be a hub if its regional betweenness centrality was 2 *SD* higher than the mean betweenness centrality of the network. The hubs were quantified based on the AUC of the betweenness centrality in the specified density range.

### Longitudinal Graph Theoretical Analyses

Longitudinal graph analysis was performed with the Graph Analysis Toolbox, version 1.4.1 (Amidi et al., 2017), using the following procedure: first, networks were normalized by the mean network strength, and the following global network metrics were quantified for the normalized networks at each time point: betweenness centrality, normalized clustering coefficient, normalized path length, small-worldness, global efficiency, local efficiency, and modularity. These network metrics were then extracted for further analyses with general linear models (see below Statistical Analyses).

### Statistical Analyses

For the baseline analyses (controls vs. TRD patients, order 0 vs. order 1 TRD patients) group comparisons on demographic variables (i.e., age) were performed with *t* tests for continuous variables, and *χ*^2^ analyses for categorical variables (i.e., gender). Within the group of TRD patients only, a repeated-measures ANOVA was conducted on HDRS scores and longitudinal graph metrics with Order (2: aiTBS > sham or sham > aiTBS) as between-subject factor and time (3: T1-T3) as within-subject factor. An exploratory analysis was also performed within responders versus nonresponders to examine changes in graph metrics with time, using a 2 (responder vs. nonresponder) × 3 (time points) repeated-measures ANOVA. Finally, Pearson product correlation coefficients were calculated within each Order (subgroups of TRD patients), between (a) the change in modularity (calculated as the difference score T_2_-T_1_), for which there was a significant order × time interaction effect, and (b) change in depression scores, that is, difference in HDRS scores between T_1_ and T_2_ (delta HDRS score T_2_-T_1_). The *p* values reported for correlations were uncorrected for multiple comparisons with a statistical threshold of *p* < .05. These analyses should be considered exploratory.

## RESULTS

### Clinical and Demographic Characteristics of the Subjects

As shown in Supporting Information Table S1 (Caeyenberghs et al., 2018), there were no significant differences in age (*t*_(104)_ = −1.231, *p* < 0.221) and gender (*χ*^2^ = 1.844, *p* < 0.226) between TRD patients and control subjects. In addition, the results revealed no significant differences in age (*t*_(44)_ = 0.815, *p* < 0.419), gender (*χ*^2^ = 0.199, *p* < 0.754), or pretreatment HDRS scores (*t*_(44)_ = 0.626, *p* < 0.534) between the two groups of TRD patients.

### Behavioral Results

Repeated-measures ANOVA showed a significant decrease of HDRS scores over time, *F*(2, 78) = 32.21, *p* < 0.001. Post hoc paired samples *t* tests showed significant reductions in HDRS scores between T_1_ and T_2_ (*p* < 0.001), and between T_2_ and T_3_ (*p* < 0.001), indicating that the HDRS score at T_2_ (mean = 21.76, *SD* = 5.65) was lower than T_1_ (mean = 17.79, *SD* = 6.26), and the HDRS score at T_3_ (mean = 14.56, *SD* = 6.87) was lower compared with T_2_. There was no significant effect of the order of treatment (sham > aiTBS vs. aiTBS > sham; *p* < 0.154) and no significant interaction effect (*p* < 0.308). Defining treatment response as a reduction of 50% from baseline HDRS scores, we found 11 responders (27%) at T_3_ in the TRD group.

### Baseline Group Differences in the Structural Connectome

We investigated (baseline) between-group differences in global network measures, comparing the AUC for these network measure curves (density range of 0.10:0.01:0.20). Both groups showed a small-world organization of the structural brain network expressed by a normalized clustering coefficient > 1, normalized path length ≈ 1, and small-world index > 1. The structural network of the TRD patients showed a significantly shorter normalized path length (*p*_*AUC*_ < 0.01) compared with the healthy control group. Also, the structural brain networks in TRD patients were characterized by a lower assortativity (*p*_*AUC*_ < 0.035) compared with controls. Group effects were absent in the other global network metrics, including global efficiency (*p*_*AUC*_ = 0.325), normalized clustering coefficient (*p*_*AUC*_ = 0.943), small-worldness (*p*_*AUC*_ = 0.737), local efficiency (*p*_*AUC*_ = 0.223), modularity (*p*_*AUC*_ = 0.460), and clustering coefficient (*p*_*AUC*_ = 0.261).

Direct comparison of the nodal graph metrics (nodal degree, local efficiency, clustering coefficient, and betweenness centrality) revealed no significant group differences after FDR correction. Results of the nodal analyses using an exploratory threshold of *p* < 0.05 are reported in Supporting Information (Caeyenberghs et al., 2018).

Finally, our qualitative analysis of the hub distribution using nodal betweenness centrality revealed that both groups exhibited seven hubs including the bilateral precuneus, bilateral superior frontal gyri, right superior parietal gyrus, left thalamus proper, and brainstem.

### Structural Network Alterations with aiTBS Stimulation

The 2 × 3 repeated-measures ANOVAs revealed a significant order × time interaction effect for modularity, *F*(2, 78) = 3.30, *p* < 0.042. However, no significant main effects of order, *F*(1, 39) = 1.301, *p* < 0.261, or time *F*(2, 78) = 0.477, *p* < 0.622, were found. Post hoc *t* test showed a significant reduction in modularity (i.e., less modular network configuration) from T_1_ to T_2_ in the order 1 group (aiTBS > sham) (*p* < 0.013), but not in the order 0 group (sham > aiTBS). No significant main effects or significant interaction effects were found for the other global network metrics.

### Structural Network Alterations in Responders

We observed a marginal significant responder × time interaction effect on betweenness centrality, *F*(2, 78) = 3.07, *p* < 0.052. The main effects of responder, *F*(2, 39) = 0.002, *p* < 0.964, and time, *F*(2, 78) = 1.558, *p* < 0.217, were not significant. Post hoc tests revealed marginally increased values of betweenness centrality in the group of responders at T_3_ compared with T_1_ (*p* < 0.072).

### Correlations Between Changes in the Structural Connectome and Changes in Depression Severity Scores

The analyses of correlations between the significant change in modularity from T_1_ to T_2_ and the changes in depression scores on the HDRS between T_1_ and T_2_ showed no direct associations (order 1 group [aiTBS > sham]: *r* = 0.208, *p* < 0.379; order 0 group [sham > aiTBS]: *r* = −0.032, *p* < 0.889).

## DISCUSSION

This study addressed the broad issue of whether graph theory and its metrics can be used to map the clinical effects of accelerated brain stimulation protocols on major depression. Specifically, ours was the first study to explore aiTBS-induced changes in the structural connectome of patients with TRD by using a well-controlled clinical trial design. Results showed immediate reductions in depression severity symptoms. Moreover, our graph theoretical analyses revealed modularity changes after 4 days of active stimulation, which suggests decreased functional segregation of the patients’ structural brain networks. This is consistent with our clinical findings in this cohort, where depression severity scores further declined 2 weeks after the aiTBS treatment protocol (Duprat et al., 2016).

### Clinical Findings

The positive impact of accelerated stimulation paradigms on depression severity is consistent with our own earlier studies (Baeken et al., 2013; Duprat et al., 2016) and others work in related psychiatric disorders, such as unipolar depression (Hadley et al., 2011; Holtzheimer et al., 2010) and suicidal patients (Desmyter et al., 2016; George et al., 2014). Although, in the present study the observed reduction in depressive symptoms was unrelated to active or sham stimulation, as revealed by a nonsignificant interaction effect between order and time for the HDRS scores (see also limitations section). The clinical analysis of the Hamilton scale (i.e., defining treatment response as a reduction of 50% from baseline HDRS scores) showed for the 41 included patients at the end of the 2-week study protocol (T3) 11 as clinical responders (27%), with only 7 in remission (17%) (defined as a HDRS scorer ≤ 7). Importantly, 2 weeks after the iTBS trial at T4 the amount of clinical responders (*n* = 17) mounted up to 41%. Twelve patients were here also considered in remission (29%). These observations indicate delayed clinical responses to aiTBS treatment. It is important to investigate whether the change in depressive scores meets the criteria for a clinically important difference, besides analyzing statistically significant differences.

### Baseline Connectome Analyses

In this study, we also investigated white matter networks of TRD patients and healthy controls by using diffusion MRI tractography and graph theoretical approaches. Although small-world properties were present for both the control and TRD group, the topological architecture of the structural networks was significantly altered in patients with TRD. First, normalized path length, which is a measure of functional integration (i.e., ability to rapidly combine specialized information from distributed brain regions; Rubinov & Sporns, 2010) was altered in TRD. Notably, normalized clustering coefficient, which is a measure of functional segregation (i.e., the ability of specialized processes to occur within highly interconnected groups of brain regions), was not affected in TRD patients. Therefore, our results suggest a preservation of the efficiency of local information transfer and processing and an impairment of global integration, likely to reflect a reduced competence in information exchange between distant brain areas. This is also supported by the lower assortativity we found in TRD patients. Second, compared with controls, our TRD patients showed lower network assortativity. In assortative networks, nodes with many connections tend to be connected to other nodes with many connections, and nodes with low connections are linked to other low-connection nodes (Newman, 2002). When network hubs are abnormally clustered and connected to low-degree nodes, assortativity drops and the structural network is less efficiently wired (Newman, 2002). Abnormalities of assortativity similar to those we have found in TRD have been described in patients with multiple sclerosis (Kocevar et al., 2016; Rocca et al., 2016).

In short, our global network analysis of TRD networks suggests a reduction in the balance between network segregation and integration and a loss of efficiency in information exchange between both close and distant regions. This is not in contradiction to former research where it has been reported that network dysfunctions may contribute to cognitive and affective abnormalities in MDD (Kaiser et al., 2015). However, mixed findings in global network metrics have been obtained in previous diffusion MRI studies examining whole-brain white matter networks in depression. For example, Bai et al. (2012) showed comparable disrupted global properties of structural networks, including reduced network strength and increased path length in remitted geriatric depression (*N* = 35). Conversely, studies by Korgaonkar et al. (2014) and Qin et al. (2014) found no significant group differences on graph measures in patients with MDD. Differences in type of depression, parcellation scheme (the automated anatomical labeling atlas, Desikan–Killiany atlas), tractography algorithm (deterministic, probabilistic), and analysis methods (network-based statistical analysis vs. graph theoretical analyses) are likely to account for differences between ours and previous results.

### aiTBS Influences Modularity in TRD Patients

Our longitudinal graph analyses revealed modularity changes after 4 days of real stimulation. Specifically, we observed a less modular network configuration in the order 1 group (aiTBS > sham) from T_1_ to T_2_. Modularity is a ubiquitous property of complex, large-scale brain networks. Modularity implies that the network is composed of a set of modules each comprising nodes that are densely connected to each other and sparsely connected to nodes in other modules (Newman & Girvan, 2004). It is possible that these transient changes in (modular) network configuration may be necessary for improvements in depressive symptoms in TRD. Our finding here also complements other experimental studies of learning and behavioral plasticity that show modularity to be predictive of cognitive effort and learning success (Bassett et al., 2011; Bola & Sabel, 2015; Kitzbichler et al., 2011; Stevens et al., 2012). For example, a MEG study by Kitzbichler et al. (2011) showed a less modular network configuration during the performance of an effortful 2-back verbal working memory task in healthy adults (*N* = 13).

Important to note, we observed the network effect in one group only (active stimulation first). We could not replicate our modularity findings in the second group (sham stimulation first). Although, a marginal significant interaction effect for modularity was observed between T_2_ and T_3_, the decreased modularity was only valid for the time interval T_1_-T_2_. We suggest that a carryover effect has biased our connectome results of our clinical trial. To overcome this issue, one should leave sufficient time (months) between the active and the sham aiTBS treatment or vice versa, which will be difficult to accept on ethical grounds, leaving TRD patients without proper treatment.

### Relationship Between Improvements in Depressive Symptoms and Changes in the Structural Connectome

No correlations were found between changes on depression severity and the degree of change of modularity. We observed nonsignificant correlations between change in the HDRS and change in modularity in the TRD subgroups (order 0, order 1). In other words, network changes did not correspond directly with clinical improvements, which may have been due to nonspecific neural responses to brain stimulation. Another explanation is that the accelerated brain stimulation protocol may have triggered changes in brain structure, but not necessarily in a way that covaried significantly with ratings of depression. Negative findings may also suggest that the network metrics affected by brain stimulation may not underpin clinical changes. Nonlinear modeling techniques may further clarify relationships between connectome and clinical changes with brain stimulation. Last, it could also be that the current scanning assessment was too short to detect modularity changes, as additional significant clinical improvement was observed 2 weeks after the aiTBS treatment (Duprat et al., 2016).

The observed reduction in depressive symptoms was unrelated to active or sham stimulation. This result is consistent with previous studies revealing clinical improvements after sham stimulation (Duecker, de Graaf, Jacobs, & Sack, 2013; Duecker & Sack, 2015; Opitz et al., 2015), suggesting by some authors that this is part of its effect (Razza et al., 2018). Although our sham procedure involved a specially designed placebo coil completely similar to the real one, which did not induce any electric fields in the human cortex, this procedure is not a pure reproduction of real rTMS, given the possible differences in skin sensations. However, clear-cut sham rTMS procedures are not available yet (Baeken et al., 2014). Nonspecific effects (e.g., TRD patients receiving uncustomary attention during the trial) may also contribute to clinical improvements after sham stimulation. Further research is necessary to investigate the effects of sham stimulation protocols.

The above caveats aside, to our knowledge this is the first study providing evidence of structural connectome changes in response to brain stimulation in TRD. Indeed, earlier research has shed some light as to how accelerated rTMS paradigms influenced local neurobiological changes in TRD patients, with changes in subgenual functional connectivity (Baeken et al., 2014, 2017), metabolism (Baeken et al., 2015), the reward system (Duprat et al., 2016), and in local GABAergic inhibitory neurotransmission (Baeken, Lefaucheur, & Van Schuerbeek 2017). Nevertheless, our current findings substantiate our former assumptions that brain changes related to clinical outcome is already present after only 4 days of stimulation. In the future, we suggest using graph theoretical analysis not only to understand the effect of brain stimulation on brain networks, but also to fine-tune brain stimulation protocols that can target a network of brain areas rather than single brain regions.

## AUTHOR CONTRIBUTIONS

Karen Caeyenberghs: Conceptualization; Formal analysis; Supervision; Writing – original draft; Writing – review & editing. Romain Duprat: Investigation; Project administration; Writing – review & editing. Alexander Leemans: Formal analysis; Methodology; Software; Writing – review & editing. Hadi Hosseini: Formal analysis; Methodology; Software; Writing – review & editing. Peter Wilson: Writing – review & editing. Debby Klooster: Formal analysis; Writing – review & editing. Chris Baeken: Conceptualization; Formal analysis; Funding acquisition; Investigation; Project administration; Writing – review & editing.

## FUNDING INFORMATION

Chris Baeken, Concerted Research Action of Ghent University, Award ID: BOF16/GOA/017. Karen Caeyenberghs, Australian Catholic University (http://dx.doi.org/10.13039/501100000990), supported by an National Health and Medical Research Council Career Development Fellowship and an ACURF Program grant by the Australian Catholic University (ACU). Chris Baeken, Ghent University Multidisciplinary Research Partnership, Award ID: The integrative neuroscience of behavioral control. Peter Wilson, Australian Catholic University (http://dx.doi.org/10.13039/501100000990), Award ID: ACURF Program grant.

## TECHNICAL TERMS

Treatment-resistant depression: Depressed patients who do not respond to mainstream treatment, e.g., medication or therapy.
Accelerated intermittent theta burst stimulation: Noninvasive neurostimulation technique that uses repeated levels of high-frequency stimulation to excitate neurons.
Connectome: Map of neural connections in the brain.
Graph theoretical analysis: A mathematical framework for quantifying topological properties of networks.
Modularity: Subdivisions of the network in groups of nodes with many links to nodes within the group and few links to nodes outside the group.
Small-world topology: Close local clustering of connections between neighboring nodes but a short path length between any distant pair of nodes caused by relatively few long-range connections.
