# Supplementary material for: Accelerated intermittent theta burst stimulation in major depression induces decreases in modularity: A connectome analysis
Source: Netw Neurosci. 2018 Nov 1;3(1):157–72. doi: 10.1162/netn_a_00060 (PMC6372023; doi:10.1162/netn_a_00060)
Supplement: Supplementary file 1 [file netn-03-157-s001.pdf]

Caeyenberghs, K., Duprat, R., Leemans, A., Hosseini, H., Wilson, P. H., Klooster, D., & Chris Baeken, C. (2018). Supporting Information for "Accelerated intermittent theta burst stimulation in major depression induces decreases in modularity: A connectome analysis." *Network Neuroscience*, *3*(1), 157–172. https://doi.org/10.1162/netn_a_00060

Supplemental Material

Supplemental Figure 1. Overview of the design.

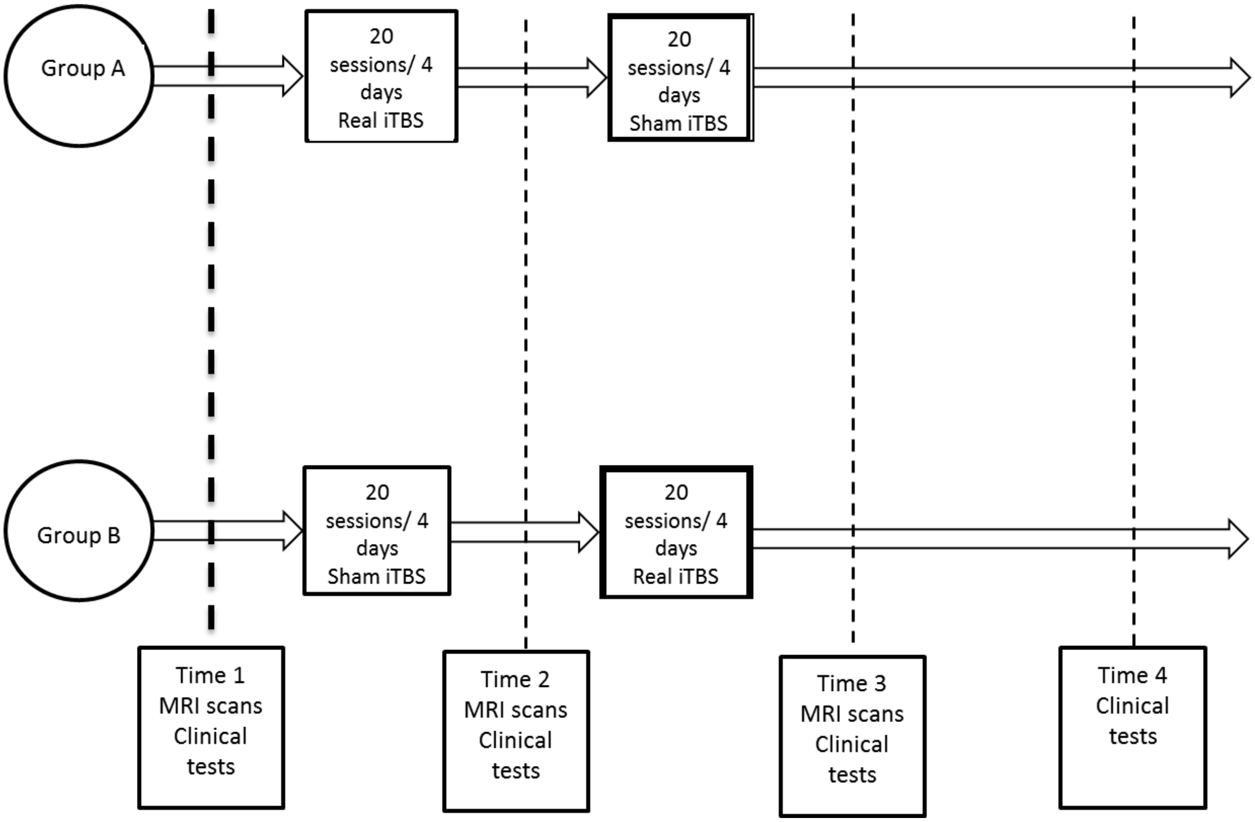

Supplemental Table 1: List of the nodes of the Desikan-Killiany atlas.

Abbreviations: ctx=cortex, lh=left hemisphere, rh=right hemisphere, DC=Diencephalon

	Region
1	lh-Cerebellum-ctx
2	lh-Thalamus-Proper
3	lh-Caudate
4	lh-Putamen
5	lh-Pallidum
6	Brainstem
7	lh-Hippocampus
8	lh-Amygdala
9	lh-Accumbens-area
10	lh-VentralDC
11	rh-Cerebellum-ctx
12	rh-Thalamus-Proper
13	rh-Caudate
14	rh-Putamen
15	rh-Pallidum
16	rh-Hippocampus
17	rh-Amygdala
18	rh-Accumbens-area
19	rh-VentralDC
20	ctx-lh-bankssts
21	ctx-lh-caudalanteriorcingulate
22	ctx-lh-caudalmiddlefrontal
23	ctx-lh-corpuscallosum
24	ctx-lh-cuneus
25	ctx-lh-entorhinal
26	ctx-lh-fusiform
27	ctx-lh-inferiorparietal
28	ctx-lh-inferiortemporal
29	ctx-lh-isthmuscingulate
30	ctx-lh-lateraloccipital
31	ctx-lh-lateralorbitofrontal
32	ctx-lh-lingual
33	ctx-lh-medialorbitofrontal
34	ctx-lh-middletemporal
35	ctx-lh-parahippocampal
36	ctx-lh-paracentral
37	ctx-lh-parsopercularis

38	ctx-lh-parsorbitalis
39	ctx-lh-parstriangularis
40	ctx-lh-pericalcarine
41	ctx-lh-postcentral
42	ctx-lh-posteriorcingulate
43	ctx-lh-precentral
44	ctx-lh-precuneus
45	ctx-lh-rostralanteriorcingulate
46	ctx-lh-rostralmiddlefrontal
47	ctx-lh-superiorfrontal
48	ctx-lh-superiorparietal
49	ctx-lh-superiortemporal
50	ctx-lh-supramarginal
51	ctx-lh-frontalpole
52	ctx-lh-temporalpole
53	ctx-lh-transversetemporal
54	ctx-lh-insula
55	ctx-rh-bankssts
56	ctx-rh-caudalanteriorcingulate
57	ctx-rh-caudalmiddlefrontal
58	ctx-rh-corpuscallosum
59	ctx-rh-cuneus
60	ctx-rh-entorhinal
61	ctx-rh-fusiform
62	ctx-rh-inferiorparietal
63	ctx-rh-inferiortemporal
64	ctx-rh-isthmuscingulate
65	ctx-rh-lateraloccipital
66	ctx-rh-lateralorbitofrontal
67	ctx-rh-lingual
68	ctx-rh-medialorbitofrontal
69	ctx-rh-middletemporal
70	ctx-rh-parahippocampal
71	ctx-rh-paracentral
72	ctx-rh-parsopercularis
73	ctx-rh-parsorbitalis
74	ctx-rh-parstriangularis
75	ctx-rh-pericalcarine
76	ctx-rh-postcentral
77	ctx-rh-posteriorcingulate
78	ctx-rh-precentral

79	ctx-rh-precuneus
80	ctx-rh-rostralanteriorcingulate
81	ctx-rh-rostralmiddlefrontal
82	ctx-rh-superiorfrontal
83	ctx-rh-superiorparietal
84	ctx-rh-superiortemporal
85	ctx-rh-supramarginal
86	ctx-rh-frontalpole
87	ctx-rh-temporalpole
88	ctx-rh-transversetemporal
89	ctx-rh-insula

Nodal analyses

Using an exploratory threshold of $p < 0.05$, the TRD group showed reduced local efficiency in the caudal (left: $p < 0.007$; right: $p < 0.031$) and rostral part (left: $p < 0.035$) of the anterior cingulate, the pars opercularis of the right inferior frontal gyrus ($p < 0.01$), the pars triangularis of the left inferior frontal gyrus ($p < 0.038$), right insula ($p < 0.047$), and right putamen ($p < 0.042$), compared with controls. An increase in local efficiency could be observed in the TRD group in the right accumbens area ($p < 0.024$), left pericalcarine gyrus ($p < 0.041$), and right cuneus ($p < 0.042$). We found decreased betweenness centrality in the left frontal pole ($p < 0.036$) in the TRD group compared with the healthy controls. For the nodal clustering coefficient, we observed reduced values in the caudal part of the anterior cingulate (left: $p < 0.036$; right: $p < 0.023$), the pars opercularis of the right inferior frontal gyrus ($p < 0.027$), the pars triangularis of the inferior frontal gyrus (left: $p < 0.048$, right: $p < 0.032$), compared with controls. Increased values of clustering coefficient could be found in the TRD group in the right accumbens area ($p < 0.041$) and right cuneus ($p < 0.045$). Finally, decreased nodal degree was found in the right bank of the superior temporal sulcus ($p < 0.045$) and right posterior cingulate gyrus ($p < 0.019$) in the TRD group. Increased nodal degree values were observed in the TRD group in the parahippocampal gyrus (left: $p < 0.014$; right: $p < 0.045$), left fusiform gyrus ($p < 0.033$), and the left lateral occipital cortex ($p < 0.015$).

Supplemental Table 2: Results of the nodal analyses using an exploratory threshold. Abbreviations: ctx=cortex, lh=left hemisphere, rh=right hemisphere, DC=Diencephalon, TRD=patients suffering from treatment-resistant depression, con=healthy control group

ROI	clustering coefficient		degree		local efficiency		betweenness centrality	
	p-value	result	p-value	result	p-value	result	p-value	result
lh-cerebellum-cx	.249		.873		.335		.926	
lh-thalamus-proper	.317		.898		.338		.851	
lh-caudate	.497		.954		.856		.994	
lh-putamen	.937		.643		.810		.894	
lh-pallidum	.223		.083		.690		.118	
brain-stem	.085		.581		.205		.998	
lh-hippocampus	.845		.177		.736		.585	
lh-amygdala	.204		.437		.174		.366	
lh-accumbens-area	.642		.429		.496		.409	
lh-ventralDC	.184		.371		.247		.520	
rh-cerebellum-cx	.791		.926		.935		.290	
rh-thalamus-proper	.368		.477		.167		.662	
rh-caudate	.307		.981		.268		.496	
rh-putamen	.120		.905		.042	TRD< con	.51	
rh-pallidum	.381		.393		.285		.778	
rh-hippocampus	.271		.310		.312		.295	
rh-amygdala	.917		.564		.881		.351	
rh-accumbens-area	.041	TRD>con	.358		.024	TRD> con	.516	
rh-ventralDC	.370		.634		.338		.769	
cx-lh-bankssts	.511		.895		.512		.270	
cx-lh-caudalanteriorcingulate	.036	TRD<con	.341		.007	TRD< con	.342	
cx-lh-caudalmiddlefrontal	.482		.241		.331		.927	
cx-lh-corpuscallosum	.548		.446		.763		.770	
cx-lh-cuneus	.171		.157		.509		.222	
cx-lh-entorhinal	.970		.122		.940		.609	
cx-lh-fusiform	.407		.033	TRD> con	.129		.512	
cx-lh-inferiorparietal	.648		.676		.846		.707	
cx-lh-inferiortemporal	.078		.793		.137		.100	
cx-lh-isthmuscingulate	.914		.376		.564		.155	
cx-lh-lateraloccipital	.854		.015	TRD> con	.121		.612	
cx-lh-lateralorbitofrontal	.274		.387		.587		.321	

cx-lh-lingual	.367		.172		.161		.433	
cx-lh-medialorbitofrontal	.365		.183		.585		.083	
cx-lh-middletemporal	.359		.392		.282		.888	
cx-lh-parahippocampal	.537		.014	TRD> con	.254		.275	
cx-lh-paracentral	.305		.989		.253		.462	
cx-lh-parsopercularis	.876		.929		.870		.754	
cx-lh-parsorbitalis	.426		.600		.684		.780	
cx-lh-parstriangularis	.048	TRD<con	.343		.038	TRD< con	.330	
cx-lh-pericalcarine	.068		.915		.041	TRD> con	.208	
cx-lh-postcentral	.306		.232		.578		.168	
cx-lh-posteriorcingulate	.719		.375		.729		.669	
cx-lh-precentral	.892		.297		.733		.709	
cx-lh-precuneus	.72		.112		.621		.169	
cx-lh-rostralanteriorcingulate	.141		.094		.035	TRD< con	.211	
cx-lh-rostralmiddlefrontal	.874		.519		.921		.988	
cx-lh-superiorfrontal	.550		.165		.793		.060	
cx-lh-superiorparietal	.615		.723		.583		.636	
cx-lh-superiortemporal	.345		.452	TRD< con	.255		.522	
cx-lh-supramarginal	.661		.700		.604		.501	
cx-lh-frontalpole	.922		.059		.569		.036	TRD<con
cx-lh-temporalpole	.907		.190		.673		.101	
cx-lh-transversetemporal	.816		.618		.977		.593	
cx-lh-insula	.945		.592		.986		.141	
cx-rh-bankssts	.243		.045		.533		.069	
cx-rh-caudalanteriorcingulate	.023	TRD<con	.672		0.031	TRD< con	.664	
cx-rh-caudalmiddlefrontal	.772		.755		.799		.904	
cx-rh-corpuscallosum	.421		.194		.333		.297	
cx-rh-cuneus	.045	TRD>con	.608		.042	TRD> con	.960	
cx-rh-entorhinal	.236		.454		.210		.486	
cx-rh-fusiform	.496		.528		.342		.112	
cx-rh-inferiorparietal	.167		.074		.515		.266	
cx-rh-inferiortemporal	.229		.276		.290		.568	
cx-rh-isthmuscingulate	.672		.415		.713		.121	
cx-rh-lateraloccipital	.785		.738		.899		.825	
cx-rh-lateralorbitofrontal	.143		.525		.125		.693	
cx-rh-lingual	.900		.21		.689		.336	
cx-rh-medialorbitofrontal	.536		.941		.531		.692	
cx-rh-middletemporal	.919		.894		.816		.781	

cx-rh-parahippocampal	.237		.045	TRD> con	.102		.698	
cx-rh-paracentral	.840		.537		.914		.612	
cx-rh-parsopercularis	.027	TRD<con	.404		.010	TRD< con	.781	
cx-rh-parsorbitalis	.101		.506		.064		.655	
cx-rh-parstriangularis	.032	TRD<con	.805		.087		.784	
cx-rh-pericalcarine	.150		.106		.104		.121	
cx-rh-postcentral	.130		.090		.367		.155	
cx-rh-posteriorcingulate	.587		.019	TRD< con	.700		.642	
cx-rh-precentral	.167		.898		.067		.280	
cx-rh-precuneus	.753		.240		.492		.527	
cx-rh-rostralanteriorcingulate	.873		.180		.963		.443	
cx-rh-rostralmiddlefrontal	.993		.798		.868		.847	
cx-rh-superiorfrontal	.483		.330		.182		.695	
cx-rh-superiorparietal	.814		.361		.875		.559	
cx-rh-superiortemporal	.934		.883		.429		.285	
cx-rh-supramarginal	.475		.869		.356		.940	
cx-rh-frontalpole	.786		.149		.276		.390	
cx-rh-temporalpole	.977		.283		.799		.406	
cx-rh-transversetemporal	.672		.411		.740		.794	
cx-rh-insula	.083		.156		.047	TRD< con	.147	
